# Enhancement of Chromatin and Epigenetic Reprogramming in Porcine SCNT Embryos—Progresses and Perspectives

**DOI:** 10.3389/fcell.2022.940197

**Published:** 2022-07-11

**Authors:** Werner Giehl Glanzner, Mariana Priotto de Macedo, Karina Gutierrez, Vilceu Bordignon

**Affiliations:** Department of Animal Science, McGill University, Sainte Anne de Bellevue, QC, Canada

**Keywords:** pig, cloning, SCNT, embryo development, chromatin, epigenetics, histone acetylation, histone methylation

## Abstract

Over the last 25 years, cloned animals have been produced by transferring somatic cell nuclei into enucleated oocytes (SCNT) in more than 20 mammalian species. Among domestic animals, pigs are likely the leading species in the number of clones produced by SCNT. The greater interest in pig cloning has two main reasons, its relevance for food production and as its use as a suitable model in biomedical applications. Recognized progress in animal cloning has been attained over time, but the overall efficiency of SCNT in pigs remains very low, based on the rate of healthy, live born piglets following embryo transfer. Accumulating evidence from studies in mice and other species indicate that new strategies for promoting chromatin and epigenetic reprogramming may represent the beginning of a new era for pig cloning.

## Introduction

**FIGURE 1:**
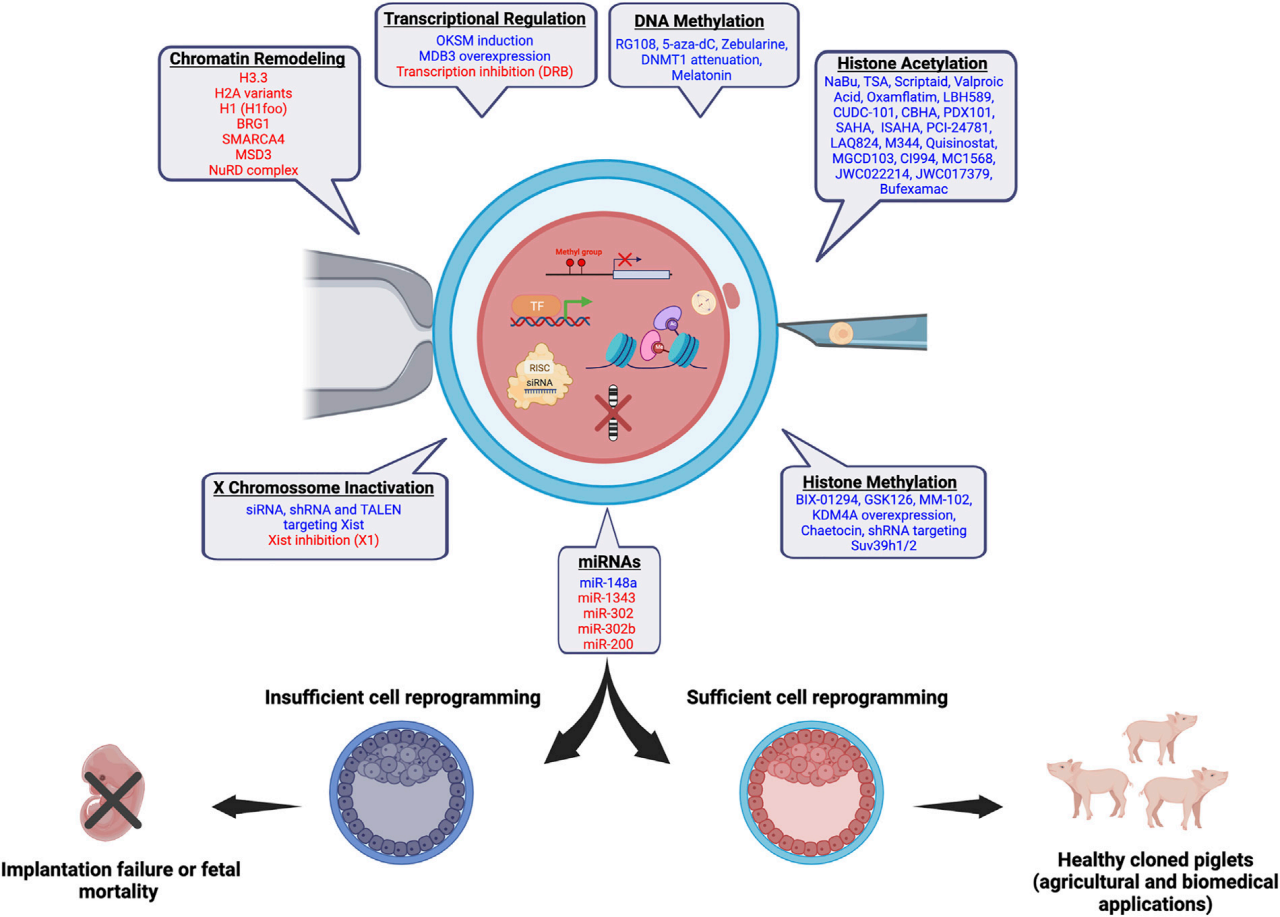
Chromatin and epigenetic reprogramming impacting SCNT efficiency in pigs. In blue, chromatin/epigenetic modifiers that have been previously shown to play a role on development of pig SCNT embryos and cloning efficiency are listed. In red, modifiers that are believed to have a beneficial effect on the development of pig SCNT embryos based on studies in other species are listed. The figure was created with BioRender.com.

The transfer of somatic cell nuclei to enucleated oocytes has proved that differentiated cells can be reverted to a totipotent state (Wilmut et al., 1997), and has helped to attain a better understanding of cell differentiation and reprogramming through chromatin and epigenetic mechanisms (Matoba and Zhang, 2018; de Macedo et al., 2021). The SCNT technology has also been applied to clone animals for different purposes, including the preservation of endangered species, replication of companion and production animals, and creation of transgenic and gene-edited animals for agricultural and biomedical purposes (Bordignon, 2019). Pigs are important for both food production and biomedical research (Gutierrez et al., 2015). Thus, pigs with unique traits and genomes of agricultural and biomedical importance have been created by SCNT, nonetheless, with low and variable efficiency. In addition of ameliorating technical aspects (e.g., oocyte maturation, enucleation, activation, embryo culture, cell fusion, cell cycle coordination), researchers have identified some roadblock mechanisms for cell reprogramming and tested new approaches to facilitate chromatin and epigenetic remodeling in SCNT embryos (Figure 1). These approaches may not only facilitate the erasure of repressive epigenetic marks that are incompatible with cell reprogramming but may also enhance the acquisition or reestablishment of specific marks required for normal embryo development and success of SCNT. This article highlights the recent progress in understanding and improving SCNT efficiency in pigs.

### Chromatin Remodeling

Successful development of SCNT embryos depends on the reestablishment of cell totipotency, which relies on changes in chromatin structure, epigenetic marks, and transcriptional profile. Nucleosomes’ positioning and occupancy affect chromatin function by either facilitating or restricting DNA access to transcription factors (TFs) and other regulators, which impacts both the reacquisition of cell totipotency following SCNT and cloning efficiency (Matoba and Zhang, 2018). Nucleosome occupancy seems to be rapidly, but likely insufficiently, reprogrammed in SCNT embryos. In pig SCNT embryos, nucleosome occupancy 10 h after nuclear transfer was lower in promoter sequences but higher in coding sequences, compared to nuclear donor cells, indicating a reprogramming process (Tao et al., 2017). Chromatin accessibility in mouse SCNT embryos was reprogrammed to a similar state of fertilized zygotes within 12 h, except for reprogramming resistant regions enriched for histone three lysine nine trimethylation (H3K9me3) (Djekidel et al., 2018).

The incorporation of maternally-derived histones facilitates chromatin remodeling in SCNT embryos. The histone H3 is replaced by the maternally-derived H3.3 in bovine (Wang et al., 2020) and mouse (Wen et al., 2014a) SCNT embryos. This switch is involved in the activation of pluripotency genes such as the Octamer-binding transcript factor (*Oct-4* or *Pou5f1*) and Nanog homeobox (*Nanog*) (Wen et al., 2014b), and reduction of epigenetic barriers for cell reprogramming, such as H3K27me3 and H3K9me3 (Wen et al., 2014b; Wang et al., 2020). In murine SCNT embryos, H2A and H2A.Z were replaced by the H2A.X variant (Nashun et al., 2011). H2A.X participates in the DNA damage response, and has been negatively correlated with embryo quality (Bohrer et al., 2013). The macro H2A variant, which is involved in the repression of pluripotency genes and considered a barrier for cell reprogramming (Gaspar-Maia et al., 2013), was also stripped from the chromatin after SCNT in mice (Chang et al., 2010). In addition, somatic variants of the linker histone H1 are reprogrammed after nuclear transfer (Bordignon et al., 1999), and are replaced by the oocyte-specific variant H1foo in SCNT embryos (Teranishi et al., 2004; Yun et al., 2012). Although H1foo′s role in chromatin reprogramming in SCNT embryos remains poorly understood, induction of *H1foo* expression along with the pluripotency factors *Oct-4*, SRY-Box Transcription Factor 2 (*Sox2*) and Kruppel Like Factor 4 (*Klf4*) improved the efficiency of mouse induced pluripotent cells (iPSCs) production (Kunitomi et al., 2016).

Chromatin relaxation, which is induced by remodeling complexes such as the transcription activator Brahma-related gene 1 (*BRG1* or *SMARCA4*), a member of the SWItch/sucrose nonfermentable (SWI/SNF) complex, represents another important feature for attaining cell totipotency and normal embryo development (Hansis et al., 2004; Egli and Eggan, 2010; Glanzner et al., 2017). The methyl-CpG-binding domain protein 3 (MBD3), a core component of the nucleosome remodeling and deacetylation (NuRD) complex, is also essential for embryo development (Hendrich et al., 2001). The NuRD complex regulates nucleosome occupancy and recruitment of TFs (Bornelov et al., 2018). Lower *MBD3* expression was detected in pig SCNT embryos compared to control embryos, and its overexpression in SCNT embryos improved blastocyst formation and total cell number (Wang et al., 2019) (Table 1).

**TABLE 1:** Impact of different modifiers of chromatin or epigenetic marks on development of SCNT embryos and pig cloning efficiency.

Chromatin/Epigenetic target	Modifier	Concentration/Duration	Target cell	Main outcomes^a^	References
Transcriptional regulation	OKSM ectopic expression	After OKSM induction iPSc-like cells were used for SCNT	Fibroblasts, Mesenchymal cells	Increased blastocyst rates and embryo cell numbers	Song et al. (2014b); Kim et al. (2019)
DNA methylation/Chromatin function	*MBD3* overexpression	10 ng/μl (∼10 pL/embryo) of the pcDNA3.1 plasmid expressing pig MBD3	Embryos	Increased blastocyst rates (from 7.4 to 20.3%) and embryo cell numbers, decreased DNA methylation, and increased *NANOG*, *OCT-4* and *LINE1* expression	Wang et al. (2019)
DNA methylation	RG108	5 nM-20 µM/1–48 h^b^	Nuclear donor cells (fibroblasts)	Increased blastocyst rates, and expression of DNA and histone methylation regulators	Diao et al., (2013); Zhai et al. (2018)
DNA methylation	Zebularine	5 nM/1–24 h^b^	Embryos and nuclear donor cells (fibroblasts)	Increased blastocyst rates, lowered global DNA methylation, and improved expression of epigenetic regulators and pluripotency genes	Diao et al. (2013); Taweechaipaisankul et al. (2019b)
DNA methylation	5-aza-dC	5 nM–0.5 µM/1–96 h^b^	Embryos and nuclear donor cells (fibroblasts)	Increased blastocyst rates and embryo cell numbers, improved DNA methylation levels and expression of epigenetic regulators and pluripotency genes	Diao et al. (2013); Huan et al. (2013); Kumar et al. (2013); Huan et al. (2015a); Huan et al. (2015b)
DNA methylation	siRNA targeting *DNMT1*	50 nM in cells and 20 µM in embryos	Embryos and nuclear donor cells (fibroblasts)	Increased blastocyst rates, improved DNA methylation patterns, and increased expression of *OCT-4*, *THY1* and pluripotency genes	Huan et al., (2015b); Song et al., (2017)
DNA methylation	Melatonin	100 nM/During IVC	Embryos	Increased blastocyst rates (from ∼20 to ∼31%) and embryo cell numbers, reduced apoptosis, improved DNA methylation of *OCT-4*, *H19*, *IGF2* and *THY1*, and upregulated expression of EGA and pluripotency genes	Qu et al. (2020)
Histone acetylation	Sodium Butyrate (NaBu)	1 mM/4–12 h^b^ for embryos 1 mM/96 h for cells	Embryos or nuclear donor cells (fibroblasts)^b^	Increased blastocyst rates and embryo cell numbers, regulated *DNMTs* mRNA levels in embryos. In donor cells: increased apoptosis, affected cell cycle, and deregulated gene expression (2 mM or higher)	Mohana Kumar et al. (2007); Das et al. (2010); Liu et al. (2012); Diao et al. (2013); Kumar et al. (2013)
Histone acetylation	Trichostatin A (TSA)	5–50 nM/10–24 h^b^	Embryos or nuclear donor cells (fibroblasts)^b^	Increased blastocyst rates and embryo cell numbers, upregulated pluripotency and developmental related genes, decreased *DNMT1* mRNA and DNA methylation, reduce apoptosis, produced pregnancies to term and improved cloning efficiency	Zhang et al. (2007); Li et al. (2008); Cervera et al. (2009); Yamanaka et al. (2009); Martinez-Diaz et al. (2010); Zhao et al. (2010); Chawalit et al. (2012); Cong et al. (2013); Diao et al, (2013); Luo et al. (2015); Jeong et al. (2021)
Histone acetylation	Scriptaid	500 nM/12–15 h for embryos	Embryos or nuclear donor cells (fibroblasts)^b^	Increased blastocyst rates and embryo cell numbers, reduced embryo apoptosis, DNA methylation and *DNMT1* expression, upregulated pluripotency and developmental related genes, increased *miR-152* expression, improved the derivation of ES-like cells, produced pregnancies to term and improved cloning efficiency	Zhao et al. (2009); Zhao et al. (2010); Diao et al. (2013); Liang et al. (2015); Whitworth et al. (2015); Lee et al. (2016); Rissi et al. (2016); Zhang et al. (2017b); No et al. (2018); Ongaratto et al. (2020)
Histone acetylation	Valproic Acid	1–8 mM/14–24 h^b^	Embryos	Increased blastocyst rates and embryo cell numbers, increased *OCT-4* mRNA, produced pregnancies to term and improved cloning efficiency	Miyoshi et al. (2010); Huang et al. (2011); Kim et al. (2011); Miyoshi et al. (2016); Sun et al. (2017)
Histone acetylation	Oxamflatin	150 nM or 1 μM/9 or 14–16 h^b^	Embryos	Increased blastocyst rates and embryo cell numbers, decreased mRNA levels of *DNMT1* and DNA methylation, upregulated *OCT-4* mRNA, produced pregnancies (not to term)	Park et al. (2012b); Hou et al. (2014); Mao et al. (2015)
Histone acetylation	LBH589	50 nM/24 h	Embryos	Increased blastocyst rates (from 10.1 to 32.5%) and embryo cell numbers, produced pregnancies (not to term)	Jin et al. (2013)
Histone acetylation	CUDC-101	1 μM/24 h	Embryos	Increased blastocyst rates (from 9.5 to 19%), produced pregnancies (not to term)	Jin et al. (2014a)
Histone acetylation	CBHA	2 μM/24 h	Embryos	Increased blastocyst rates (from 12.7 to 26.5%), and mRNA levels of *OCT-4*, *CDX2* and *IGF2*, produce pregnancies to term, but did not improve cloning efficiency	Song et al. (2014a)
Histone acetylation	PDX101	0.5 μM/24 h	Embryos	Increased blastocyst rates (from 10.6 to 25.7%) and embryo cell numbers, produced pregnancies (not to term)	Jin et al. (2015)
Histone acetylation	SAHA	7.5–10 μM/6–16 h^b^ for embryos 1.5–6 μM/24–72 h^b^ for cells	Embryos or nuclear donor cells (fibroblasts)^b^	Embryo treatment: increased blastocyst rates, upregulated lysosome, steroid biosynthesis and glycosaminoglycan degradation, downregulated KEGG pathways, modulated *OCT-4* and *HDAC1* mRNA levels, and produced pregnancies to term. Cell treatment: increased blastocyst rates, and improved gene expression in SCNT embryos	Whitworth et al. (2015); Sun et al. (2017); Kim et al. (2018); Sun et al. (2020)
Histone acetylation	ISAHA	1 μM/14–16 h	Embryos	Increased embryo cell numbers, upregulated some lysosome, steroid biosynthesis and terpenoid backbone biosynthesis, produced 75% pregnancy rates with development to term	Whitworth et al. (2015)
Histone acetylation	PCI-24781	0.5 nM/6 h	Embryos	Increased blastocyst rates (from 10.2 to 25.2%), decreased apoptosis, produced pregnancies (not to term)	Jin et al. (2016)
Histone acetylation	LAQ824	100 nM/24 h	Embryos	Increased blastocyst rates (from 14.2 to 29.9%), embryo cell numbers, decreased DNA methylation, improved mRNA levels of developmental genes	Jin et al. (2017a)
Histone acetylation	M344	5 μM/6 h	Embryos	Increased blastocyst rates (from 10.9 to 25.1%), reduced apoptosis, produced pregnancies to term	Jin et al. (2017b)
Histone acetylation	Quisinostat	10–100 nM/24 h^b^	Embryos	Increased blastocyst rates, decreased DNA methylation, improved mRNA levels of pluripotency/developmental genes, produced pregnancies to term	Jin et al. (2017c); Taweechaipaisankul et al. (2019a)
Histone acetylation	MGCD0103	0.2 μM/6 h	Embryos	Increased blastocyst rates (from 10.5 to 21.2%), produced pregnancies (not to term)	Jin et al. (2017d)
Histone acetylation	CI994	10 μM/24 h	Embryos	Increased blastocyst rates (from 11.4 to 21.9%), decreased apoptosis, increased *OCT-4* and *SOX2* expression	Jin et al. (2018)
Histone acetylation	MC1568	10 μM/12 h	Embryos	Increased blastocyst rates (from 20.6 to 33.2%) and embryo cell numbers, increased mRNA levels of *OCT-4*, *SOX2*, *NANOG* and *CDX2*	Wang et al. (2018)
Histone acetylation	JWC022214	2 μM/22–24 h	Embryos	Increased blastocyst rates (from 36.9 to 59.7%)	Siriboon et al. (2018)
Histone acetylation	JWC017379	2–4 μM/22–24 h	Embryos	Increased blastocyst rates (from 50 to 79.4%) and primary outgrowths of SCNT-derived ES-like cells	Siriboon et al. (2018)
Histone acetylation	Bufexamac	20 μM/oocyte IVM	Host oocytes during maturation	Increased blastocyst rates (from 16.3 to 25%) and mRNA levels of *OCT-4* and *CDX2*	Sun et al. (2021)
Histone acetylation/DNA methylation	Combination of RG108 and Scriptaid	200 µM RG108 and 100 nM Scriptaid/17–19 h	Embryo	Increased blastocyst rates (from 19.1 to 29.3%) and embryo cell numbers, rescued methylation patterns of *H19* and *XIST*, increased *NANOG* expression	Xu et al. (2013)
Histone acetylation/DNA methylation	Combination of 5-aza-dC and TSA	50 nM–0.025 μmol/L (TSA); 2.5 nM–0.01 μmol/L (5-aza-dC)/1–24 h (TSA); 1–72 h (5-aza-dC)^b^	Nuclear donor cells (fibroblasts)	Increased blastocyst rate, decreased DNA methylation levels	Diao et al. (2013); Ning et al. (2013)
Histone methylation	BIX-01294 (*G9A* inhibitor)	50 nM/14–16 h	Embryos	Increased blastocysts rates (from 16.4 to 23.2%), modulated mRNA levels of pluripotency and epigenetic related genes, produced pregnancies to term and improved cloning efficiency	Huang et al. (2016)
Histone methylation	GSK126 (*EZH2* inhibitor)	0.75 µM/48 h in cells and 0.1 µM/24 h in embryos	Embryos and nuclear donor cells (fibroblasts)	Increased blastocysts rates (from 20.2 to 31.3%)	Xie et al. (2016)
Histone methylation	MM-102 (H3K4 HMT inhibitor)	75 µM/72 h	Embryos	Increased blastocysts rates (from 8.9 to 25.7%) and embryo cell numbers, modulated DNA methylation, and mRNA levels of pluripotency and epigenetic related genes	Zhang et al. (2018b)
Histone methylation	*KDM4A* overexpression	*In vitro* synthetized mRNA coding *KDM4A* (500 ng/μL) injected (10 pL) 5 h post-activation	Embryos	Increased blastocysts rates (from 20.9 to 32.2%) and embryo cell numbers	Weng et al. (2020)
Histone methylation	Chaetocin	0.5–10 nM/6 or 24 h or during 6 h in the 4-cell stage^b^	Embryos	Increased blastocysts rates, hatching rates and embryo cell numbers, upregulated pluripotency genes, downregulated *DNMTs*, reduced DNA methylation and apoptosis, and regulated developmental related genes	Jeong et al. (2020); Weng et al. (2020); Jeong et al. (2021)
Histone methylation	shRNA targeting *SUV39H1/2*	MOI-600 of a lentivirus containing shRNA targeting *SUV39H1/2*. SCNT performed after 72 h post-incubation	Nuclear donor cells (fibroblasts)	Increased blastocyst rates (from 15.5 to 29.9%) and embryo cell numbers. In the donor cells: modulated mRNA levels of cell cycle-related genes and epigenetic modifiers	Cao et al. (2021)
Histone methylation/Histone acetylation	BIX-01294/Scriptaid	25 nM BIX-01294 + 250 nM Scriptaid/14–16 h	Embryos	Increased blastocysts rates (from 14 to 23.7%)	Huang et al. (2016)
Histone methylation/Histone acetylation	BIX-01294/TSA	1 µM BIX-01294 + 50 nM TSA/24 h	Embryos	Increased blastocysts rates (from ∼20 to ∼44%) compared to control but not to TSA alone, decreased global DNA methylation in the trophectoderm	Cao et al. (2017)
Histone methylation/Histone acetylation	Chaetocin/TSA	0.5 nM Chaetocin +50 nM TSA/24 h	Embryos	Increased blastocyst rates (from ∼21 to ∼34%), hatching rates, and embryo cell numbers, reduced apoptosis and DNA methylation, modulated EGA and imprinting related genes	Jeong et al. (2021)
miRNA	*miR-148a* overexpression	MOI-50 of a lentivirus containing miRNA-148a sequence were transfected in cells/SCNT was performed with puromycin selected cells	Nuclear donor cells (fibroblasts)	Increased blastocysts rates (from 14.3 to 20.8%), hatching rates, and embryo cell numbers, reduced DNA methylation, increased H3K9 acetylation levels in 2-cell embryos, increased *OCT-4* and *NANOG* mRNA.	Wang et al. (2017)
X chromosome inactivation	siRNA targeting *XIST*	10 pL of 5 μM; injected 6–7 h post-activation/Effective KD until 16 cell-stage	Embryos	Increased embryo cell numbers, produced pregnancies to term and improved cloning efficiency	Zeng et al. (2016)
X chromosome inactivation	TALEN targeting *XIST*	20 μg of DNA template and 10 μg of *XIST* TALEN electroporated into the porcine fetal fibroblasts	Nuclear donor cells (fibroblasts)	Increased blastocyst rates (from 25.4 to 36.4%) and embryo cell numbers, produced pregnancies to term and improved cloning efficiency	Ruan et al. (2018)
X chromosome inactivation	shRNA targeting *XIST*	10 pL of 5 ng/μL plasmid; injected into the cytoplasm of each blastomere of 2 cell-embryo	Embryos	Increased blastocyst rates (from 15.5 to 28.7%) and embryo cell numbers, increased expression of X-linked genes	Yang et al. (2019)

a Main outcomes considering results observed in all the publications. The same outcomes are not necessarily observed by all researchers or reported in all publications.

b Different concentrations, periods of incubation or target cells (nuclear donor cells or embryos) have been used in the different publications.

### Transcriptional Regulation

Proper regulation of TFs is crucial for reprograming cell totipotency and successful development of SCNT embryos (Table 1). Overexpression of the iPSC inducing factors *OCT-4*, *SOX2*, *KLF4* and *c-MYC* (OSKM) in porcine cells prior to nuclear transfer improved blastocyst rates and quality of SCNT embryos (Song Z. et al., 2014; Kim et al., 2019). The co-expression of OKSM and the estrogen-related receptor B (*ESRRB*), which is abundantly expressed in pig embryos (Yu et al., 2021), improved iPSCs production by regulating pluripotency factors (Shi et al., 2020). In addition, pig iPSCs overexpressing *ESRRB* showed higher potential for trophectoderm differentiation when injected into 8-cell stage embryos (Yu et al., 2021).

The impact of *OCT-4* on development of SCNT embryos has been investigated in multiple different species. In pigs, expression of *OCT-4* and *OCT-4* related genes, such as Calcium binding and coiled-coil domain 2 (*NDP52l1*), and Developmental pluripotency associated 2, three and 5 (*DPPA2*,*3*,*5*), was lower in SCNT than control embryos (Lee et al., 2006). The Double homeobox (Dux) transcription factor and its upstream regulators Dppa2 and four have been identified as major regulators of embryonic genome activation (EGA) (Eckersley-Maslin et al., 2019). Overexpression of *Dux* in mouse SCNT embryos improved development and normalized EGA transcripts, comparable to levels of control embryos produced by fertilization (Yang et al., 2020). Despite of these relevant findings in other species, the impact of manipulating EGA regulators has not been studied in porcine SCNT embryos.

Proper reprogramming to totipotency in SCNT embryos can be hampered by both lack of gene activation and failure to inactivate the transcriptional memory of somatic nuclei following nuclear transfer (Ng and Gurdon, 2005; Matoba et al., 2014). The transcriptional memory of somatic cells was more efficiently reprogrammed in SCNT embryos that cleaved early and produced higher blastocyst rates than late cleaving embryos (Liu et al., 2020). In addition, attenuation of the transcriptional memory for 15 h after nuclear transfer, using the inhibitor of RNA polymerase II, 5,6-Dichlorobenzimidazole 1-β-d-ribofuranoside (DRB), improved gene expression in bovine SCNT embryos and increased blastocyst cell numbers (Rissi et al., 2018). However, more studies are needed to determine the impact of transcriptional inhibition following SCNT on cloning efficiency in pigs.

### DNA Methylation

DNA methylation is usually associated with transcriptional silencing. Substantial DNA demethylation occurs during early embryo development but remethylation happens at later stages (Reik et al., 2001; Ivanova et al., 2020). In SCNT embryos, timely DNA demethylation and remethylation should occur to enable proper gene expression and cell reprogramming. However, donor cells are usually highly methylated and demethylation seems to be incomplete in SCNT embryos (Cao et al., 2020). In mice, several genes important for early development, including *Dppa2/4*, Oocyte-specific homeobox 6 (*Obox6*) and TEA domain transcription factor 4 (*Tead4*), failed to activate in SCNT embryos due to abnormal DNA methylation (Cao et al., 2020). In pigs, 4-cell SCNT embryos presented higher DNA methylation levels and expression of the DNA methyl transferase 1 (*DNMT1*) than control embryos (Deshmukh et al., 2011; Song et al., 2017). Attenuation of *DNMT1* in nuclear donor cells 36 h prior to nuclear transfer decreased DNA methylation levels of *OCT-4*, *NANOG* and *SOX2*, increased their expression at EGA and blastocyst stages, and improved blastocyst rates of pig SCNT embryos (Song et al., 2017). A similar effect was observed by attenuating *DNMT1* expression after nuclear transfer, which normalized the methylation status of *OCT-4* and the Thy-1 cell surface antigen (*THY1*), a fibroblast marker, promoting *OCT-4* activation and *THY1* silencing in pig SCNT embryos (Huan et al., 2015b). Overexpression of *MBD3* in pig SCNT embryos corrected methylation levels of *NANOG*, *OCT-4* and long interspersed nuclear elements (LINEs), and increased blastocyst rates and quality (Wang et al., 2019).

Treatment of nuclear donor cells with 5-aza-2′-deoxycytidine (5-aza-dC), which incorporates onto DNA during replication leading to hypomethylation by inhibiting *Dnmt1* action (Stresemann and Lyko, 2008), has been another strategy tested to improve SCNT efficiency. This approach increased the development and quality of pig SCNT embryos (Diao et al., 2013; Kumar et al., 2013), and enhanced transcript levels of *DNMT1, two* and *3* (Kumar et al., 2013; Huan et al., 2015b). Treatment of pig SCNT embryos with 5-aza-dC for 24 h after nuclear transfer increased embryo development, which was associated with a decrease in methylation levels of *NANOG* and an increase in the expression of *NANOG* and *SOX2* (Huan et al., 2015a). Treatment with other inhibitors of DNA methyltransferases, including Zeburaline and RG108, also increased development of pig SCNT embryos (Zhai et al., 2018; Taweechaipaisankul et al., 2019b). A combined treatment of RG108 with Scriptaid, a histone deacetylase inhibitor, rescued defective methylation patters in the imprinted gene *H19*/insulin-like growth factor 2 (*IGF2*), and increased development of pig SCNT embryos (Xu et al., 2013). Moreover, melatonin was shown to favor DNA methylation reprogramming, and expression of imprinted, pluripotency and EGA related genes in pig SCNT embryos (Qu et al., 2020) (Table 1). Overexpression of the Ten-eleven translocation 3 (*TET3*) enhanced SCNT efficiency in bovine (Zhang et al., 2020), and goats (Han et al., 2018), however, this has not been investigated in pigs yet.

### Histone Acetylation

Pig oocytes have high levels of acetylated histones H3 and H4, especially at the germinal vesicle (GV) stage (Endo et al., 2005). Histone acetylation levels are controlled by acetyltransferases (HAT) and deacetylases (HDAC) enzymes (Endo et al., 2011). Pig oocytes express several HDACs, which regulate histone acetylation levels during oocyte maturation (Endo et al., 2008). The acetylation status of the lysine nine in the histone 3 (H3K9ac) has been proposed as a biomarker for embryo outcome, since it can be altered by culture conditions and it affects EGA and embryo development (Rollo et al., 2017). Normal maturation of pig oocytes is affected by HDACs inhibition (Jin Y.-X. et al., 2014; Huang et al., 2021).

There is consensus, based on studies conducted in different species, that development of SCNT embryos is improved by treatment with HDAC inhibitors (HDACi). Several HDACi molecules were shown to enhance pig SCNT efficiency and cell reprogramming. This includes PDX101 (Belinosat), LBH589 (Panobinostat), CI994, CUDC-101, MGCD0103, MC1568, JWC022214 (HDACi-14), JWC017379 (HDACi-79), PCI-24781, m-carboxycin- namic acid bishydroxamide (CBHA), Suberoylanilide hydroxamic acid (SAHA) 4-iodo-SAHA (ISAHA), Quisinostat, Bufexamac, M344, LAQ824, Oxamflatin, Sodium butyrate (NaBu), Valproic acid, and the more commonly used Scriptaid, and Trichostatin A (TSA) (Table 1). HDACi have also been associated with other molecules, including DNA methylation modifying agents (Table 1).

Although HDACi treatment has become an important component of SCNT protocols, the mechanism by which it promotes cell reprogramming has not been fully elucidated. In addition of enabling a more permissive chromatin state and improving gene expression by increasing acetylation levels, HDCAi treatment may also facilitate DNA damage repair in embryos (Bohrer et al., 2014; Wang et al., 2015). There is evidence that the effect of HDACi treatment in SCNT embryos is influenced by the differentiation state of the nuclear donor cell (Kishigami et al., 2006; Martinez-Diaz et al., 2010), and also by cell cycle interactions between the host cytoplast and the nuclear donor cell at the time of nuclear transfer (Rissi et al., 2016).

### Histone Methylation

Histone methylation is crucial for cell reprograming and normal embryo development by controlling important embryo features such as EGA, cell differentiation and DNA damage response (Dahl et al., 2016; Qin et al., 2016; Glanzner et al., 2017; Glanzner et al., 2018; Jambhekar et al., 2019; Rissi et al., 2019; Glanzner et al., 2020). In the context of cell reprogramming in SCNT embryos, interest in histone methylation has gained more emphasis after an increase of 3.4-fold in blastocyst rates (88 vs. 26%) and 8.7-fold development to term (8.7 vs. 1%) was obtained by expressing the demethylase *Kdm4d* in mouse SCNT embryos (Matoba et al., 2014). In addition, *Kdm4d* expression in human SCNT embryos improved the establishment of SCNT-derived embryonic stem cell cultures (Chung et al., 2015). The impact of manipulating the expression of specific demethylases on development of SCNT embryos has been studied in several species including sheep (Zhang Y. et al., 2018), cattle (Liu et al., 2018; Zhou et al., 2019), and swine (Rissi et al., 2019; Glanzner et al., 2020). Among the histone methylation markers studied, H3K9me3 and H3K27me3 have been identified as the most important barriers of SCNT success (Matoba et al., 2014; Xie et al., 2016). In pigs, SCNT efficiency increased by attenuating H3K9me3 levels, either by suppressing specific methyltransferases (*SUV39H1/2*, *G9A*) (Huang et al., 2016; Jeong et al., 2020; Weng et al., 2020; Cao et al., 2021), or by expressing the demethylase *KDM4A* (Weng et al., 2020). Similarly, the inhibition of the H3K27 methyltransferase *EZH2* increased pig SCNT efficiency, while the inhibition of the H3K27 demethylase *UTX* (also known as *KDM6A*) decreased the efficiency (Xie et al., 2016). There is also evidence supporting an important role for H3K4 methylation, which is normally associated with an accessible chromatin state and transcriptional activity, in the regulation of SCNT embryos (Hormanseder et al., 2017). In pigs, depletion of H3K4 methylation increased blastocyst rates and improved gene expression patterns in SCNT embryos (Zhang Z. et al., 2018).

The association of different epigenetic modifiers has been tested to improve pig SCNT efficiency. For example, increasing histone acetylation along with decreasing H3K9me3 levels (Cao et al., 2017; Jeong et al., 2021) or decreasing DNA methylation (Huang et al., 2016; Cao et al., 2017), improved gene expression patterns in SCNT embryos and pig cloning efficiency (Table 1).

### Micro RNAs

Micro RNAs (miRNAs) have important roles on cell reprogramming and regulation of normal embryo development (Blakaj and Lin, 2008; Pauli et al., 2011; O'Brien et al., 2018). miRNAs are small RNA sequences (∼22/23 nucleotides) that regulate gene functions by pairing to target mRNAs and inducing their degradation or repressing translation (Bartel, 2004; O'Brien et al., 2018). There is evidence that miRNAs are required for normal oocyte growth and maturation, and early embryogenesis in several species, including pigs (Prather et al., 2009; Kaczmarek et al., 2020). For example, deletion of the RNase III endonuclease *DICER*, which is important for the biogenesis of miRNAs, impaired mouse embryo development due to a decrease in mature miRNAs (Tang et al., 2007). During early embryo development, miRNAs regulate the silencing of transcripts that are no longer necessary for development, modulate chromatin rearrangements, and promote cell pluripotency (Tang et al., 2007; Prather et al., 2009; Pauli et al., 2011). They also contribute to the regulation of embryo implantation and embryo-maternal communication (Kaczmarek et al., 2020). Examples of miRNAs identified to have important roles on embryo gene silencing, cell pluripotency and cell differentiation include *miR-430, miR-125, miR-145* and *let-7* (Blakaj and Lin, 2008; Bartel, 2009; Pauli et al., 2011).

Studies in pigs revealed that miRNAs, including *miR-1343, miR-302, miR-302b* and *miR-200*, are involved in the acquisition and maintenance of cell pluripotency by regulating the expression of TFs, such as *SOX2* and *OCT-4* (Ma et al., 2014; Qiao et al., 2019; Xie et al., 2019). However, the impact of miRNAs during cell reprogramming and development of pig SCNT embryos has not been extensively explored. In mice, *miR-125b* was identified as a crucial factor for cell reprogramming in SCNT embryos by regulating the expression of methyltransferases (e.g., *Suv39h1*) that control H3K9me levels and chromatin accessibility (Zhang J. et al., 2017). In pigs, overexpression of *miR-148a* in nuclear donor cells downregulated *DNMT1* expression and increased blastocyst rates and total cell numbers in SCNT embryos (Wang et al., 2017) (Table 1). Treatment of pig SCNT embryos with the HDACi Scriptaid attenuated *DNMT1* expression and H3K9me3 levels, as well as increased miR-152 expression, suggesting a link between miRNAs, *DNMT1*, and histone methylation and acetylation (Liang et al., 2015). However, more studies are required to dissect the regulation and cross talk between miRNAs and epigenetic regulation in pig SCNT embryos.

### X Chromosome Inactivation

Long non-coding RNAs **(**lncRNAs) are known to regulate several biological processes such as chromatin function, signaling pathways, and mRNA stability (Statello et al., 2021). The X-inactive specific transcript gene produces a lncRNA (*Xist*), which is a major regulator of X chromosome inactivation (XCI) (Penny et al., 1996). During normal embryo development, XCI is required for compensation of X-linked genes between males and females. In mouse embryos, inactivation of the paternally-derived X chromosome starts at the early cleavage stages of development (Okamoto et al., 2011). In pig embryos, XCI is random and only observed in the late epiblast stage, as evidenced by the reduction of biallelic expression of X-linked genes and increase in H3K27me3 levels (Ramos-Ibeas et al., 2019).

Proper reprogramming of the X chromosome seems a critical component of SCNT efficiency (Table 1). Downregulation of X-linked genes due to the ectopic expression of *Xist* was detected in mouse SCNT embryos (Matoba et al., 2011). Similarly, *XIST* and X-linked genes were aberrantly expressed in pig SCNT embryos (Park C.-H. et al., 2012; Mao et al., 2015), and SCNT fetuses having abnormal development (Yuan et al., 2014; Ruan et al., 2018). Inactivation of *XIST* in nuclear donor cells or its attenuation in SCNT embryos improved blastocyst rates and cloning efficiency in mice and pigs (Matoba et al., 2011; Zeng et al., 2016; Ruan et al., 2018; Yang et al., 2019). A recent study demonstrated that the small molecule X1 can target a specific motif of *Xist* and blocks initiation of XCI (Aguilar et al., 2022). This may represent a new alternative for preventing early inaction of X chromosome in SCNT embryos, however, more studies are needed to evaluate efficiency and toxicity in pig embryos.

### Concluding Remarks and Future Perspectives

Recent progress in xenotransplantation of pig organs, along with the evolution of technologies for editing the pig genome has expanded interest in the production of cloned pigs by SCNT. Since it was proven that SCNT can make somatic cells regain totipotency, researchers in the field have been attempting to increase the efficiency of this reproductive method, but the progress has been modest. While *in vitro* development to the blastocyst stage of SCNT embryos is often similar to fertilized embryos, development to term and rate of alive cloned piglets remain low, confirming that not all chromatin functions regulating development have been reset properly. Cumulating evidence, mainly from mouse studies, pointed out that epigenetic marks, such as DNA and histone methylation, as well as histone acetylation, transcription factors, and non-coding RNAs, can all affect cell reprogramming and SCNT efficiency. Moreover, efforts to modulate these factors in SCNT embryos to mimic fertilized embryos by using molecules, or either attenuating or overexpressing genes, has shown encouraging results that improved not only the blastocyst rate and quality, but also development to term of cloned animals (Figure 1). In addition, some attempts taken to modulate multiple factors have further ameliorated mouse cloning efficiency, suggesting this route could be explored to improve SCNT protocols in other species, including pigs. For example, inhibition of HDAC and transcription along with either attenuation or expression of specific modulators of histones or DNA methylation may likely improve porcine SCNT efficiency. It is worth highlighting however, that there are fundamental differences between species in the regulation of early development, including the timing of EGA and first cell lineage specification, which should be taken in consideration when translating findings from one species to another.

## Data Availability

The original contributions presented in the study are included in the article/supplementary material, further inquiries can be directed to the corresponding author.
